# Toxicité cutanée à l'erlotinib

**DOI:** 10.11604/pamj.2013.15.47.2579

**Published:** 2013-06-08

**Authors:** Hayat Bourra, Badreddine Hassam

**Affiliations:** 1 Service de Dermatologie, CHU Ibn Sina, Université Med V, Souissi, Rabat, Maroc

**Keywords:** Erlotinib, Tarceva, inhibiteurs à l'Epidermal Growth Factor, éruption acnéiforme, Erlotinib, Tarceva, à l'Epidermal Growth Factor inhibitors, acneiform eruption

## Image en médecine

L'Erlotinib, commercialisé sous le nom de Tarceva est un anticancéreux appartenant au groupe des inhibiteurs spécifiques de l'activité tyrosine kinase du récepteur du facteur de croissance épidermique “EGFr”. Il est indiqué dans les formes localement avancées ou métastatiques du cancer bronchique non à petites cellules et du cancer du pancréas métastatique après échec d'au moins une ligne de Chimiothérapie. L'utilisation de ces molécules confronte les thérapeutes à la gestion de nouvelles toxicités notamment cutanées. Parmi les effets indésirables les plus fréquents nous retrouvons l'éruption acnéiforme, à type de papulo-pustules folliculaires monomorphes prurigineuses sans comédons, mimant une acné induite. Cette éruption présente une bonne valeur prédictive de réponse au traitement. Les traitements proposés en fonction du grade clinique, sont les antibiotiques per os à action anti-inflammatoire (doxycycline) et des traitements locaux alternant dermocorticoïdes, antibiotiques locaux (érythromycine, clindamycine) ou métrodinazole topique. L'éruption est réversible à l'arrêt du traitement et parfois spontanément malgré sa poursuite. Des séquelles de type télangiectasies ou hyperpigmentation peuvent survenir. Nous rapportons l'observation d'un patient âgé de 52 ans, suivi pour carcinome pulmonaire non à petites cellules métastatique en 2ème ligne de chimiothérapie à base d'Erlotinib 150mg/j, a présenté 6 semaines après introduction d'Erlotinib des lésions cutanées papulo pustuleuses à prédominance inflammatoire réalisant un aspect sycosis like, des lésions érythémato squameuses des plis nasogéniens, une sécheresse buccale et chéilite. Le bilan bactériologique était stérile. Devant l'aspect clinique, le patient a été classé grade 2 selon le National Cancer Institute, mis sous cyclines 100mg/j et poursuite de l'Erlotinib avec bonne évolution.

**Figure 1:**
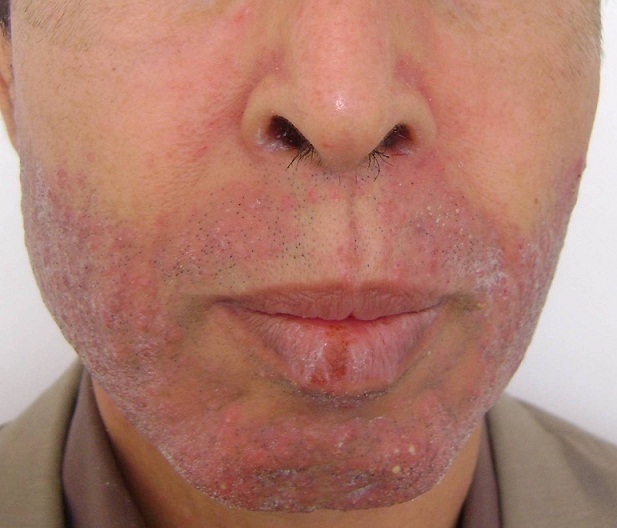
Éruption papulo-pustuleuse folliculaire et séborrhéique du visage associée à une chéilite

